# The diagnostic value of 11q13 amplification and protein expression in the detection of nodal metastasis from oral squamous cell carcinoma: a systematic review and meta-analysis

**DOI:** 10.1007/s00428-015-1719-6

**Published:** 2015-02-07

**Authors:** Rob Noorlag, Pauline M. W. van Kempen, Inge Stegeman, Ron Koole, Robert J. J. van Es, Stefan M. Willems

**Affiliations:** 1 Department of Oral and Maxillofacial Surgery, University Medical Center Utrecht, Utrecht, Netherlands; 2 Department of Otorhinolaryngology, University Medical Center Utrecht, Utrecht, Netherlands; 3 Department of Pathology, University Medical Center Utrecht, H4.241, Heidelberglaan 100, 3584 CX Utrecht, The Netherlands; 4 Brain Center Rudolf Magnus, University Medical Center Utrecht, Utrecht, Netherlands

**Keywords:** 11q13, Oral cavity, Nodal metastasis, Systematic review, Meta-analysis

## Abstract

Despite improvements in both diagnostic and therapeutic strategies, the prognosis of oral squamous cell carcinoma (OSCC) has not changed significantly over the last decades. Prognosis of OSCC particularly depends on the presence of nodal metastasis in the neck. Therefore, proper determination of the nodal status is pivotal for appropriate treatment. Unfortunately, current available imaging techniques (magnetic resonance imaging or ultrasound even with fine needle aspiration of suspected lymph nodes (LNs)) fail to detect occult nodal disease accurately. Clinicians in head and neck oncology urgently need new diagnostic tools to reliably determine the presence of nodal metastasis of the neck. Gain of the chromosomal region 11q13 is one of the most prominent genetic alterations in head and neck cancer and is associated with poor prognosis and metastasis. The aim of this systematic review and meta-analysis was to determine the diagnostic value of either 11q13 amplification or amplification/protein overexpression of individual genes located on 11q13 to detect nodal metastasis in OSCC. A search was conducted in Pubmed, EMBASE, and Cochrane, and 947 unique citations were retrieved. Two researchers independently screened all articles and only 18 were found to meet our inclusion criteria and were considered of sufficient quality for meta-analysis. Pooled results of those show that both amplification of *CCND1* and protein overexpression of cyclin D1 significantly correlate with lymph node metastasis (LNM) in OSCC. In addition, amplification of *CCND1* shows a negative predictive value of 80 % in the detection of LNM in early stage OSCCs which are clinically lymph node negative although this evidence is sparse and should be validated in a larger homogeneous cohort of T1-2 OSCC.

**Electronic supplementary material:**

The online version of this article (doi:10.1007/s00428-015-1719-6) contains supplementary material, which is available to authorized users.

## Introduction

Head and neck cancer is a heterogeneous group of malignancies and the sixth most common malignancy worldwide [1]. Approximately one third of all head and neck squamous cell carcinoma (HNSCC) consists of oral squamous cell carcinoma (OSCC). Despite improvements in both diagnostic and therapeutic strategies over the past decades, 5-year overall survival rate has not improved significantly and remains poor with on average 50–60 % [1, 2]. The prognosis of OSCC is largely determined by the presence or absence of lymph nodal metastasis (LNM). Therefore, proper determination of the nodal status of the neck is pivotal. Unfortunately, current available imaging techniques such as magnetic resonance imaging (MRI) or even ultrasound with fine needle aspiration of suspected lymph nodes fail to detect the presence of nodal metastasis accurately; 30 to 40 % of patients with clinically lymph node negative neck have occult nodal metastasis and will develop nodal disease if the neck is left untreated [3]. This urges for better diagnostic tools to detect regional metastasis more accurately. Ultimately, this will result in a better and a more individualized treatment of the neck in patients with OSCC.

To improve diagnostics of nodal status in OSCC, new techniques such as molecular diagnosis and tumor profiling are promising [3]. Amplifications and deletions of chromosomal regions are genetic alterations and both driving forces in carcinogenesis of several malignancies [4]. Gain of the chromosomal region 11q13 has been established as one of the most prominent (36 %) genetic alterations in head and neck cancer and is associated with poor prognosis [5]. Recent research identified 11q13.3 as the most frequently amplified gene region: It contains several potential driver genes such as *CCND1*, *CTTN*, *FADD*, *FGF19*, and *ORAOV1* [6]. A recent review with meta-analysis indicated that immunohistochemical overexpression of cyclin D1 located on 11q13 (protein of gene *CCND1*) correlated both with the presence of nodal metastasis and a worse survival in an Asian population with OSCC [7]. For amplification of *CCND1* and amplification or overexpression of any of the other genes located on chromosome 11q13.3, the diagnostic value in determining nodal metastasis in OSCC is unclear, and no comprehensive review has been conducted yet. The relationship between amplification or overexpression of the 11q13 region or genes located on 11q13 and the detection of LNM in primary OSCC has been explored, and the number of papers is increasing rapidly. However, none of these biomarkers is used in current clinical practice since study results are conflicting and results of adequately designed translational studies are lacking [8–11].

Therefore, we conducted a systematic review and meta-analyses if possible, of all studies performed to date, to define the overall diagnostic value of 11q13.3 amplification or overexpression of its individual genes in the detection of LNM from OSCC.

## Material and methods

### Search strategy

We conducted a systematic search for original articles published until the 30th of April 2014 in the Pubmed, EMBASE, and Cochrane databases for original articles. Search terms used were “oral cancer,” “11q13” (or individual genes located on 11q13), and “metastasis” and their synonyms in title and abstract fields; see Supplementary Table S1. All titles and abstracts were independently screened by two authors (R.N. and P.M.W.K.) using predefined inclusion and exclusion criteria (see below). Subsequently, the full text of relevant studies was screened for a more detailed selection. Discordant judgments were resolved by consensus discussion. Reference and citation check of selected articles was performed to identify potentially missed relevant studies.

### Inclusion and exclusion criteria

For this review, full-text articles were selected on the basis of (1) correlation of 11q13 overexpression or amplification with (2) nodal metastasis in (3) patients with OSCC or HNSCC with a subgroup of OSCC, with (4) clinical or histopathological nodal status as reference standard.

Used exclusion criteria were (1) duplicate articles that contained all or some of the original publication data, (2) reviews, book chapters, cases reports, editorials, oral presentations, technical notes, and poster presentations, (3) articles which included head and neck cancer without a subgroup of OSCCs, and (4) articles in a language other than English, German, or Dutch.

### Critical appraisal and data extraction

Quality assessment of included studies was performed by critical appraisal, based on standardized criteria for diagnostic research using the QUADAS-2 tool for quality assessment of diagnostic accuracy studies [12]. Risk of bias was scored as low, high, or unknown (if the item was not mentioned in the article) based on the following items:(1) patient selection—consecutive cohort of patients, avoidance of case-control, and avoidance of inappropriate exclusions; (2) index test—researchers blinded to reference standard and pre-specified threshold; (3) reference standard—validity of reference standard and blinding for the index test; and (4) flow and timing—interval between and standardization of test and reference standard, and completeness of data. In addition, the first three items were also scored on applicability for this review: (1) patient selection—only OSCC included in study; (2) index test—dichotomized outcome with cutoff point instead of continuous outcome and useful for review question; and (3) reference standard—either histological nodal status or follow-up of an untreated neck for at least 2 years.

We extracted first author, year of publication, country, sample size, tumor location, TNM stage, distribution or average age, used antibody, investigated genes/proteins, method, and outcome from each study. Amplification or overexpression and nodal metastasis data for crosstabs were extracted from included studies. All studies with source data for a crosstab available were included in the meta-analysis. In case of insufficient data, authors were contacted to provide the source data. For complete and transparent reporting of the results of our review, we used the preferred reporting items for systematic reviews and meta-analyses (PRISMA) statement checklist [13].

### Statistical analysis

Odds ratios (ORs) were used to describe the correlation between 11q13 amplification or overexpression of its genes and nodal metastasis. Negative predictive value (NPV), positive predictive value (PPV), accuracy, sensitivity, and specificity were calculated from extracted crosstabs using the EPR-Val Toolkit Version 2 [14]. If insufficient data were available, for example, if only the *p* value mentioned in the included article was published, the study was excluded from further meta-analysis.

For meta-analysis, the conservative random effect model was used to calculate the pooled estimates, and statistical significance was determined using the Z-test [15]. Test for heterogeneity across studies was performed using both Q test and the Higgins I^2^. The Higgins I^2^ describes the proportion of inter-study variability in effect estimates that is due to heterogeneity rather than sampling error (change) and ranges from 0 to 100 %. Although distinct values are arbitrary since more factors influence heterogeneity, I^2^ values of 0, 25, 50, and 75 % are indicated as “no,” or a “low,” “moderate,” and “high” amount of heterogeneity [16, 17]. All statistical tests for meta-analyses were performed using Comprehensive Meta-Analysis 2.0 software (Biostat, Englewood, NJ), and *p* values <0.05 (two-sided) were considered statistically significant.

## Results

### Article selection

**Fig. 1:**
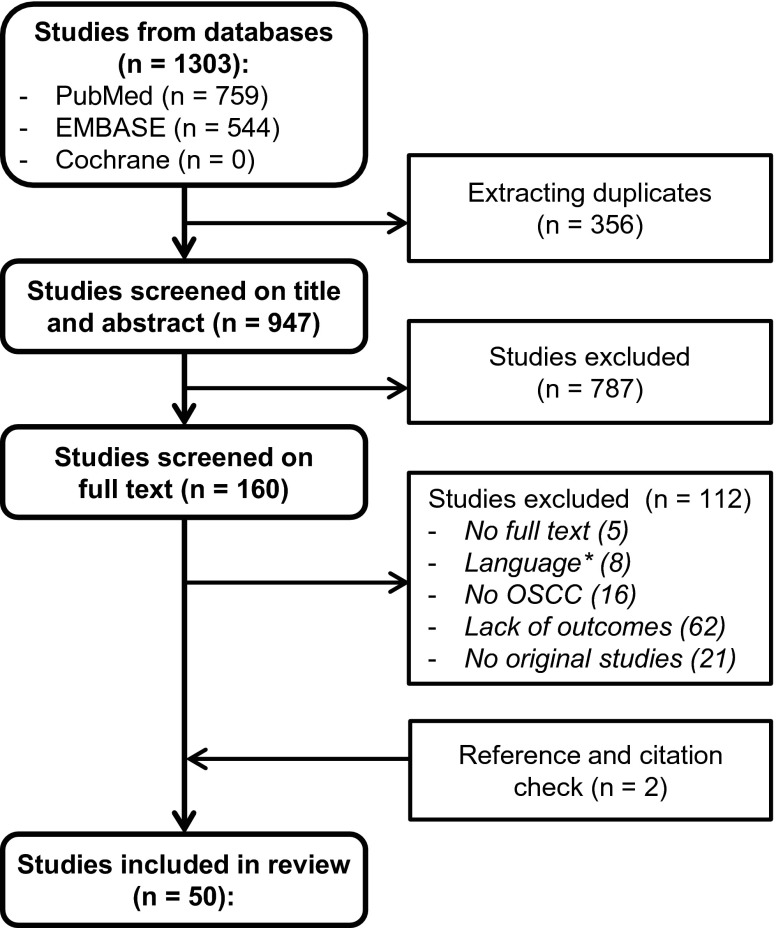
Flowchart search. * Languages: Chinese (4), Polish (2), Japanese (1) and Spanish (1)

Our search resulted in 1303 citations, 759 from PubMed and 544 from the EMBASE database. After removal of duplicates, 947 unique citations remained for screening on title and abstract. After both title and abstract screening and full text screening, original research papers were included for critical appraisal. Three (Myo, Miyamoto, and Michikawa) articles from the same institute with partly overlapping inclusion data were included [9, 18, 19]. Michikawa et al. [19] was the most recent article with the largest group of patients in which detection of *CCND1* amplification was performed in relation to LNM; however, Myo et al. [9] was the only study that performed a subanalysis in the clinically most relevant group of early OSCCs which were clinically lymph node negative. Therefore, we decided to include both studies in our review. Additionally, Miyamoto et al. was the only study who performed protein expression analysis of cyclin D1 next to CCND1 amplification in this cohort. Therefore, we decided to solely include the protein expression analyses of this study in our review and meta-analysis [18]. Reference and citation check revealed two additional papers which met our inclusion criteria; see flowchart in Fig. 1. These studies were not included in our initial search because the study of Yoshioka et al. [20] did not mention 11q13 or any of the individual genes in the tile or abstract and the study of Takahashi et al. [21] was not indexed in PubMed or EMBASE. However, Takahashi et al. [21] might have reported overlapping data with Michikawa et al. [19] as the enrolment periods overlap completely; therefore, we did exclude this article for our review and meta-analysis.

### Critical appraisal

All 50 studies that were selected for further analysis were appraised by the QUADAS-2 tool for quality assessment of diagnostic accuracy studies. They were scored on risk of bias and applicability for this review; see Table 1. Eighteen studies were found of sufficient applicability with respect to our review question [8–11, 18–33]. Nine of these studies scored all four items as low risk of bias and the other nine scored three out of four items as low risk of bias. According to the QUADAS-2 tool, the quality of these 18 articles was good or moderate, and they were included for result analysis. Nine of the included studies investigated the correlation between gene amplification and nodal metastasis; ten studied the correlation between protein overexpression and nodal metastasis; see Table 2. From 32 excluded studies, 18 studies scored moderate (three out of four items, low risk) or good (all four items, low risk) quality with respect to risk of bias. The main reasons for insufficient applicability of these studies were (1) inclusion of other head and neck subsites than oral cavity without subgroup analysis and/or (2) clinical nodal status instead of histologically proven nodal metastasis or adequate follow-up as reference standard. Fourteen studies scored bad (one or two items, low risk) on risk of bias and applicability and therefore were excluded from further analysis.

**Table 1:** Quality assessment of studies included

Year/first author	Risk of bias					Applicability concerns			
	Patient selection	Index test	Reference standard	Flow and timing		Patient selection	Index test	Reference standard	
2012 Huang	+	+	+	+	Low risk	+	+	+	Applicable for review
2011 Sugahara	+	+	+	+		+	+	+	
2011 Pathare	+	+	+	+		+	+	+	
2011 Michikawa^a^	+	+	+	+		+	+	+	
2011 Mahdey	+	+	+	+		+	+	+	
2009 Shah	+	+	+	+		+	+	+	
2005 Myo^a^	+	+	+	+		+	+	+	
2003 Miyamoto^a^	+	+	+	+		+	+	+	
2002 Takes	+	+	+	+		+	+	+	
2014 Hanken	+	+	+	−	Moderate risk	+	+	+	
2013 Yoshioka	−	+	+	+		+	+	+	
2010 Prapinjumrune	+	−	+	+		+	+	+	
2007 Maahs	+	?	+	+		+	+	+	
2005 Rodolico	+	+	+	−		+	+	+	
2002 Goto	?	+	+	+		+	+	+	
2001 Fujii	+	+	+	−		+	+	+	
1999 Bova	−	+	+	+		+	+	+	
1999 Kuo	+	?	+	+		+	+	+	
2013 Pattje	+	+	+	+	Low risk	−	+	+	Not applicable for review
2004 Do	+	+	+	+		−	+	+	
2000 Rodrigo	+	+	+	+		−	+	+	
1997 Muller^a^	+	+	+	+		−	+	+	
1997 Fortin	+	+	+	+		−	+	+	
2013 Fan	−	+	+	+	Moderate risk	−	+	+	
2013 Li	+	+	?	+		+	+	?	
2012 Rasamny	+	+	−	+		−	+	−	
2011 Das	+	+	?	+		+	+	?	
2006 Wang	+	+	?	+		+	+	?	
2005 Shiraki	+	+	−	+		+	+	−	
2005 Soni	+	+	−	+		+	+	−	
2003 Vora	+	+	?	+		−	+	?	
2000 Capaccio	−	+	+	+		−	+	+	
2000 Mineta	+	+	−^b^	+		+	+	−	
1997 Kyomoto	+	+	−	+		−	+	−	
1994 Muller^a^	−	+	+	+		−	+	+	
1994 Parise	?	+	+	+		−	+	+	
2014 Pickhard	+	?	+	?	High risk	−	−	+	
2013 Zhong	+	+	−	−		+	+	−	
2010 Yamada	−	+	?	+		+	+	?	
2009 Liu	−	+	?	−		+	+	?	
2007 Xia	−	+	?	+		+	+	?	
2006 Zhou	−	−	+	+		+	−	+	
2004 Liu	?	+	−	+		+	+	−	
2004 Chen	−	+	?	−		+	+	?	
2002 de Vicente	−	+	?	+		+	+	?	
2002 Namazie	−	+	?	−		−	+	?	
1999 Alavi	+	+	?	?		−	+	?	
1995 Meredith	?	?	?	−		−	−	?	
1995 Rubin	?	+	?	+		+	+	?	
1994 Volling	?	?	?	+		−	?	?	

Legend: +, low risk; −, high risk; ?, unclear. “Unclear” was seen as high risk of bias for determining the quality of a paper

^a^Studies with overlapping patient inclusion

^b^Corresponding author contacted, used CT/MRI as reference standard

**Table 2:** Characteristics of included studies

	First author	Year	Country	Sample size	Tumor site	TNM stage	Reference standard	Nodal metastasis ^a^	Genes	Method	Tissue	Cutoff
Gene amplification	Hanken	2014	Germany	255	Oral cavity	All	pN	46 %	CCND1	FISH	FFPE	G/C ratio >2.0
	Yoshioka	2013	Japan	25	Oral cavity	All	pN	60 %	11q13.3	CGH	FFPE	G/C ratio >1.12
	Sugahara	2011	Japan	54	Oral cavity	All	pN	41 %	11 genes in 11q13^c^	CGH	Fresh frozen	G/C ratio >1.5 in ≥3 genes
	Pathare	2011	India	97	Oral cavity	N.A.	pN	56 %	11q13	CGH	Fresh frozen	G/C ratio >1.25
	Michikawa	2011	Japan	127	Oral cavity	All	pN	42 %	CCND1	FISH	Fixed FNA	G/C ratio >1.2 and gene/cell ratio >3
	Mahdey	2011	Malaysia	50	Cheek and tongue	All	pN	54 %	CCND1	FISH	FFPE	G/C Ratio >2.0
	Prapinjumrune^b^	2010	Japan	60	Tongue	T1-2	pN or FU	43 %	FADD	RT-PCR	FFPE	G/C ratio >1.5
	Myo	2005	Japan	45	Oral cavity	cT1-2N0	pN or FU	38 %	CCND1	FISH	Fixed FNA	>20 % of 100 cells ≥3 spots
	Fujii	2001	Japan	23	Tongue	All	pN or FU	61 %	CCND1	FISH	FFPE	>20 % of 100 cells ≥3 spots
Protein overexpression	Huang	2012	Taiwan	264	Oral cavity	All	pN	48 %	CCND1	IHC	FFPE	10 % staining
	Prapinjumrune ^b^	2010	Japan	60	Tongue	T1-2	pN or FU	40 %	FADD	IHC	FFPE	29.2 % staining
	Shah	2009	India	135	Cheek and tongue	All	pN	33 %	CCND1	IHC	FFPE	10 % staining
	Maahs	2007	Brazil	45	Oral cavity	All	pN	51 %	CCND1	IHC	FFPE	1 % staining
	Rodolico	2005	Italy	97	Lower lip	Any T cN0	pN	13 %	CCND1	IHC	FFPE	1 % staining
	Miyamoto ^b^	2003	Japan	41	Oral cavity	All	pN	20 %	CCND1	IHC	FFPE	10 % staining
	Takes	2002	Netherlands	52	Oral cavity	All	pN	63 %	CCND1	IHC	FFPE	5 % staining
	Goto	2002	Japan	41	Tongue	cT1-2	pN	44 %	CCND1	IHC	FFPE	33 % staining
	Kuo	1999	Taiwan	88	Oral cavity	All	pN	60 %	CCND1	IHC	FFPE	10 % staining
	Bova	1999	Australia	147	Tongue	All	pN	23 %	CCND1	IHC	FFPE	10 % staining

*TNM* tumor-node-metastasis, *FISH* fluorescence in situ hybridization, *CGH* comparative genomic hybridization, *IHC* immunohistochemistry, *FFPE* formalin-fixed paraffin-embedded, *FNA* fine needle aspiration, *G/C* gene/chromosome

^a^Histologically proven nodal metastasis

^b^These studies correlated both gene amplification and protein overexpression with nodal metastasis

^c^TPCN2, MYEOV, CCND1, ORAOV1, FGF4, TMEM16A, FADD, PPFIA1, CTTN, SHANK2, DHCR7

### Study characteristics

In total, the selected 18 studies comprised a total of 1646 patients (range, 23–264 patients); 736 patients were included in studies correlating gene amplification with nodal status and 970 patients in studies correlating protein overexpression with nodal status. Thirteen studies were performed in Asia, three in Europe, one in Australia, and one in Brazil. Most studies included all stages of OSCC, but three studies looked specifically at early (stage I–II) cancers of which the study of Myo et al. investigated the clinically most relevant group of early OSCCs which were clinically lymph node negative [9, 26, 29]. The differences in selected study population resulted in a wide range of prevalence of histologically proven nodal metastasis (13 to 63 %). In the studies investigating the diagnostic value of gene amplification for the detection of nodal metastasis, five articles studied the diagnostic accuracy of *CCND1* amplification using fluorescence in situ hybridization (FISH) for the detection of LNM [9, 11, 19, 22, 25]. Three studies looked at amplification of 11q13 region using the combination of multiple genes with comparative genomic hybridization (CGH) [20, 23, 24], and one study looked at amplification of *FADD* using RT-PCR [26]. Besides different detection methods, also several definitions of amplification were used among these studies. In the studies investigating the diagnostic accuracy of protein overexpression of genes located at 11q13 in detection of nodal metastasis, nine articles studied immunohistochemistry (IHC) of cyclin D1 and one study of *FADD* [8, 10, 26–33]. Half of the studies used 10 % staining as cutoff value for overexpression, three studies had lower, and two studies had higher cutoff points. Most studies used a pre-specified cutoff; only Prapinjumrune et al. established overexpression as expression in the top two thirds of the study cohort (>29.2 %) [26]. Study characteristics are summarized in Table 2.

### Diagnostic value of 11q13 amplification region or individual genes located on 11q13 in detection of nodal metastasis

Table 3 shows the diagnostic accuracy of all studies correlating gene amplification of 11q13 region and nodal metastasis. Besides a wide range in prevalence of histologically proven nodal metastasis, the nine studies showed a wide range in the detected amount of amplification (26 to 72 %). Three studies showed a statistically significant correlation between amplification of 11q13 (or individual genes) and the presence of nodal metastasis in OSCC [9, 19, 23]. However, the other six studies did not find a correlation between amplification and nodal metastasis. The NPV ranged from 30 to 83 % and the PPV from 38 to 80 %. With a threshold of ≥3 spots of *CCND1* in >20 % of 100 cells under the microscope, a commonly used threshold in FISH, Myo et al. [9] found the best accuracy (82 %) and was also the only study investigating nodal metastasis in a cohort of clinically nodal negative early OSCC (cT1-2N0). Meta-analysis of the five studies correlating *CCND1* amplification by FISH with nodal metastasis revealed a statistically significant increase in risk of nodal metastasis with an odds ratio of 2.12 (95 % confidence interval (CI) 1.43–3.16), with moderate risk of heterogeneity (I^2^ = 65) [9, 11, 19, 22, 25]. Meta-analysis of the three studies correlating 11q13 amplification by CGH with nodal metastasis showed no statistically significant correlation (odds ratio 2.00 with 95 % CI 0.77–5.21), with moderate risk of heterogeneity (I^2^ = 46) [20, 23, 24]. The results are presented in forests plots in Fig. 1, and the tests of heterogeneity are shown in Table 5.

**Table 3:** Diagnostic accuracy of 11q13 amplification for nodal status in OSCC

Study	Nodal metastasis^a^	Threshold for amplification	Amplification	OR (95 % CI)	*p* value	NPV (%)	PPV (%)	AC (%)	SE	SP
Hanken et al.	117/255, 46 %	G/C ratio >2.0	69/255, 27 %	1.66 (0.95–2.90)	0.074	57	55	57	32	77
Yoshioka et al.	15/25, 60 %	G/C ratio >1.12	13/25, 52 %	1.14 (0.23–5.67)	0.870	42	62	52	53	50
Sugahara et al.	22/54, 41 %	G/C ratio >1.5 in ≥3 genes	14/54, 26 %	5.83 (1.52–22.33)	0.010	70	71	70	45	88
Pathare et al.	54/97, 56 %	G/C ratio >1.25	40/97, 41 %	1.35 (0.60–3.06)	0.473	47	60	53	44	63
Michikawa et al.	53/127, 42 %	G/C ratio >1.2 and gene/cell ratio >3	43/127, 34 %	2.78 (1.30–5.92)	0.008	67	58	64	47	76
Mahdey et al.	27/50, 54 %	G/C ratio >2.0	36/50, 72 %	1.87 (0.54–6.51)	0.327	57	58	58	78	35
Prapinjumrune et al.	13/30, 43 %	G/C ratio >1.5	13/30, 43 %	0.70 (0.16–3.05)	0.638	53	38	47	38	53
Myo et al.	17/45, 38 %	>20 % of 100 cells ≥3 spots	15/45, 33 %	20 (4.09–97.90)	<0.001	83	80	82	71	89
Fujii et al.	14/23, 61 %	>20 % of 100 cells ≥3 spots	13/23, 57 %	0.50 (0.09–2.84)	0.434	30	54	43	50	33

*OR* odds ratio, *CI* confidence interval, *G/C* gene/chromosome, *NPV* negative predictive value, *PPV* positive predictive value, *AC* accuracy, *SE* sensitivity, *SP* specificity

^a^Histologically proven nodal metastasis

### Diagnostic value of 11q13 overexpression in detection of nodal metastasis

Table 4 shows the diagnostic accuracy of all studies correlating protein overexpression of genes located on 11q13 and nodal metastasis. Most studies correlated immunohistochemical expression of cyclin D1 with nodal metastasis, except Prapinjumrune et al. who looked at FADD expression [26]. The amount of overexpression of cyclin D1 varied from 32 to 83 %. Two studies showed a significant correlation between cyclin D1 overexpression and nodal metastasis in OSCC [8, 32], Goto et al. [30] found a trend toward more metastasis in tumors with overexpression, and the other six studies found no correlation at all. The NPV ranged from 32 to 73 % and the PPV from 37 to 85 %. The diagnostic accuracy of most studies was poor, the best being 66 % in the study of Goto et al. [30] Prapinjumrune et al. also found a significant correlation between FADD expression and nodal metastasis, with a NPV of 44 % and a PPV of 83 % [26]. Two articles had insufficient data for meta-analyses [22, 29]. Although these authors were contacted by e-mail, these data were not provided.

**Table 4:** Diagnostic accuracy of 11q13 overexpression for nodal status in OSCC

Study	Nodal metastasis^a^	Threshold for overexpression	Overexpression	Primary antibody	OR (95 % CI)	*p* value	NPV (%)	PPV (%)	AC (%)	SE	SP
Huang et al.	126/264, 48 %	10 % staining	97/264, 37 %	Monoclonal, SP4	2.48 (1.48–4.15)	0.001	73	48	61	62	60
Prapinjumrune et al.	24/60, 40 %	29.2 % staining	40/60, 66 %	Monoclonal, 1/FADD	4.00 (1.14–14.09)	0.025	44	83	60	50	80
Shah et al.	44/135, 33 %	10 % staining	43/135, 32 %	Monoclonal, P2D11F11	1.35 (0.63–2.90)	0.435	70	37	59	36	70
Maahs et al.	23/45, 51 %	1 % staining	15/45, 33 %	N.A.	1.71 (0.49–6.03)	0.401	53	60	56	39	73
Rodolico et al.	13/97, 13 %	1 % staining	65/97, 67 %	Monoclonal, DCS-6	N.A.	N.A.	N.A.	N.A.	N.A.	N.A.	N.A.
Miyamoto et al.	8/41, 20 %	10 % staining	27/41, 66 %	Monoclonal, DCS-6	1.71 (0.30–9.87)	0.546	36	75	44	22	86
Takes et al.	33/52, 63 %	5 % staining	30/52, 58 %	Monoclonal, DCS-6	0.70 (0.22–2.23)	0.546	32	60	48	54	37
Goto et al.	18/41, 44 %	33 % staining	14/41, 34 %	Monoclonal, DCS-6	3.60 (0.93–13.95)	0.064	67	64	66	50	78
Kuo et al.	53/88, 60 %	10 % staining	73/88, 83 %	Polyclonal, N.A.	1.41 (0.46–4.30)	0.550	47	62	59	85	20
Bova et al.	34/147, 23 %	10 % staining	100/147, 68 %	Monoclonal, D1-GM	3.43 (1.23–9.54)	0.018	37	85	48	29	89

*OR* odds ratio, *CI* confidence interval, *G/C* gene/chromosome, *NPV* negative predictive value, *PPV* positive predictive value, *AC* accuracy, *SE* sensitivity, *SP* specificity, *N.A.* not available

^a^Histologically proven nodal metastasis

**Fig. 2:**
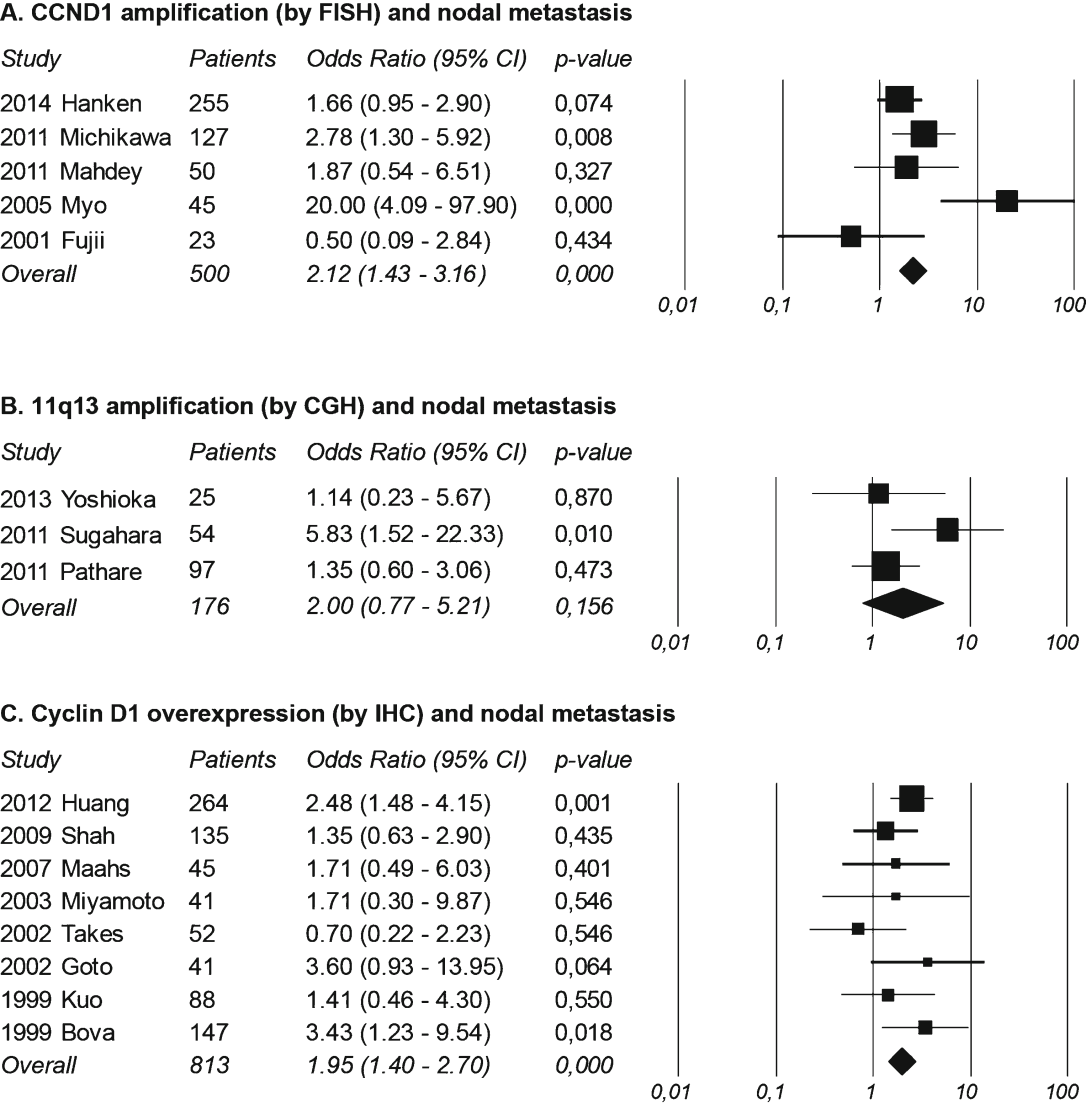
Meta-analyses of (A) *CCND1* amplification, (B) 11q13 amplification and (C) cyclin D1 overexpression and nodal metastasis using random-model method with Odds Ratio’s and 95 % CI in figures

Meta-analysis of the seven studies correlating cyclin D1 overexpression by immunohistochemistry (IHC) with the presence of nodal metastasis revealed a statistically significant increase in risk of nodal metastasis with an odds ratio of 1.95 (95 % CI 1.40–2.70), with low risk of heterogeneity (I^2^ = 1) [8, 10, 27, 28, 30–33]. See also forest plot and test of heterogeneity in Fig. 2 and Table 5.

**Table 5:** Heterogeneity in meta-analysis

Meta-analysis	Q-value	df (Q)	*p* value	I^2^
Cyclin D1 overexpression (IHC)	7.086	7	0.420	1.212
*CCND1* amplification (FISH)	11.597	4	0.021	65.509
11q13 amplification (CGH)	3.727	2	0.155	46.341

*IHC* immunohistochemistry, *FISH* fluorescence in situ hybridization, *CGH* comparative genomic hybridization

## Discussion

New diagnostic biomarkers to improve the diagnosis of nodal metastasis in patients with OSCC are pivotal for a better and more individualized treatment of the neck [3]. Amplification of 11q13 is common in head and neck cancer, and several studies showed a correlation with metastasis and poor survival. However, results vary between studies, and no coherent review has been performed at present with regard to the diagnostic value of 11q13 amplification, amplification of individual genes located on 11q13, or overexpression of its genes in the detection of nodal metastasis from oral cancer. Little is known about the NPV of these alterations, which is the most important diagnostic value to safely omit an elective treatment of the neck in patients with early OSCCO. Overall, the results of our meta-analysis show that both amplification of *CCND1* and overexpression of cyclin D1 correlate with nodal metastasis in OSCC. Furthermore, *CCND1* amplification seems to have great potential as a diagnostic biomarker for lymph node metastasis in a subgroup of clinically nodal negative OSCC although supporting evidence is still not very strong.

The strength of a systematic review depends on the quality of the search, critical appraisal, and reporting of the review. For selection of studies, we used the validated QUADAS-2 tool to judge their quality [12]. The first finding in this critical appraisal was the large number of studies which included patients with head and neck cancer originating from different subsites, or used clinical nodal status as reference standard instead of histologically proven metastasis. Discrimination of head and neck subsites is particularly relevant, because multiple studies show differences in genetic alterations, such as mutations, amplification, or deletions, between its different subsites [34–36]. As a consequence, the effects of amplification of 11q13 in any other location of the head and neck than the oral cavity cannot be extrapolated to OSCC. For this reason, we excluded all studies which included tumors other than OSCC and all studies without a separate location analysis. As mentioned earlier, the determination of nodal metastasis with imaging modalities is inaccurate in OSCC. Therefore, we excluded all studies using another reference standard than either histologically proven nodal metastasis or follow-up of the neck for at least 2 years [3].

Despite the fact that all studies analyzed included only OSCC and used histologically proven nodal metastasis as a reference standard, the correlation and diagnostic accuracy between amplification of the 11q13 region as well as overexpression of cyclin D1 and LNM still varied among the included studies. There are several possible explanations for these differences: First of all, there is heterogeneity in the stages of the included OSCCs. Most studies included all different stages of OSCC and three studies included only early stage OSCC [9, 26, 29]. Since early stages of OSCC show less genetic alterations than late stage OSCC, this could explain the stronger correlation in these three studies focused on stage 1–2 OSCC compared with the more variable correlation in studies that included all stages of OSCC [36]. Although Hanken et al. [22] found no significant differences in *CCND1* amplification between T1-2 and T3-4 OSCC, this study did not look at the correlation of *CCND1* amplification and LNM in these subgroups. Second, differences in methodological setup might explain part of the differences as the used assays, explored genes, and cutoff point for amplification varied between the 12 amplification studies. Although most protein expression studies used the same methods and explored the same genes (IHC for cyclin D1), these studies used different primary antibodies (see Table 4) and there was a wide range in the definition for overexpression (1–33 %) [8, 10, 27–33]. Third, geographical or ethnical differences may account for a different outcome. Sixteen of the included studies were carried out in Asians, three in Caucasians, and one in an ethnically mixed group, although no divergent results were observed in outcome, which is in line with an earlier review [7]. Finally, one has to realize that OSCC includes tumors arising from different oral cavity subsites such as the cheek, floor of the mouth, and oral tongue. Meanwhile, an increasing number of studies have appeared that show differences in molecular biology between these oral cavity subsites [25] (Table 2).

This meta-analysis shows a significant correlation between both cyclin D1 protein overexpression as well as *CCND1* amplification by FISH and the detection of nodal metastasis in OSCC. It is noteworthy that the strength of correlation between *CCND1* amplification and the detection of LNM might be influenced by possible overlapping data in two included studies [9, 19]. Furthermore, a recent review in an Asian population by Zhao et al. found a slightly stronger correlation between cyclin D1 overexpression and nodal metastasis. However, they used a fixed-model method in their meta-analyses and also included studies using clinical nodal status as reference standard [7]. In order to use the fixed-model method, two conditions have to be fulfilled: (1) There must be good reasons to believe that all studies are functionally identical and (2) the computed effect cannot be generalized beyond the population included in the analysis. Although the Q-test for heterogeneity was not significant for cyclin D1 overexpression, we believe that the included studies are not functionally identical due to above mentioned differences in materials and methods, and therefore, we applied the more conservative random-model method. This model allows differences in effect size between studies and therefore leads to wider confidence intervals, especially if only few studies are included in the meta-analysis [15].

Meta-analysis of 11q13 amplification by CGH showed no significant correlation with nodal metastasis, which may seem contradictory to the other results. This inconsistency may be explained as follows: First of all, two studies (Yoshioka et al. and Pathare et al.) used relatively low cutoff values for amplification compared with the other CGH study and the FISH studies; see Table 2. Second, the total sample size of this meta-analysis was small and included only 176 patients, compared with 500 patients in the *CCND1* amplification by FISH analysis and 813 patients in the cyclin D1 overexpression analysis; see Fig. 2.

For potential use as diagnostic tool in clinical decision making, the NPV of a biomarker in clinically lymph node negative OSCC is even more important than an overall correlation, since false negative results have serious consequences for the patient [37]. Unfortunately, only two studies (Myo et al. and Rodolico et al.) investigated the role of *CCND1* amplification or cyclin D1 protein overexpression in this specific subgroup, both with a significant correlation with nodal metastasis [9, 30]. Of these studies, only Myo et al. reported sufficient data to reliably extract the NPV (83 %) and PPV (80 %). These results are promising considering the pre-test probability of 38 % for a nodal metastasis but need further validation in a larger cohort. The source data of the other study unfortunately could not be obtained from contacted authors.

Although we performed a comprehensive and systematic review with transparent methods, quality check, and extraction of study results, several limitations have to be mentioned. First, the search for this review was restricted to studies published in English, German, and Dutch, which after quality check led to inclusion of 20 articles (8 articles were excluded because of the language). In comparison with the review by Zhao et al., we could have missed some articles written in Chinese [7]. Second, because of the known inconsistence between clinically and histologically proven nodal metastasis, we only included studies that explicitly mentioned “pathological” or “histological” nodal metastasis in their manuscript and we left out three articles of moderate/good quality on risk on bias (at least three out of four items, low risk; see Table 1) that were unclear about their reference standard for nodal status. Although the likelihood of introduction of bias was minimized, potentially relevant studies could have been omitted from this analysis. Third, all presented studies are based on analysis of resection specimen. To be of diagnostic value for daily clinical practice, it would be relevant to validate these findings for incisional biopsies as well. Finally, we did not stratify our meta-analyses for anatomical subsites in the oral cavity. This may have to be taken into account in future analysis since evidence is increasing that these different locations might show different molecular alterations during carcinogensis [25].

In conclusion, according to current available evidence, both amplification of *CCND1* and overexpression of cyclin D1 are potential biomarkers in the detection of LNM in OSCC. For early stage OSCC, which is the clinically most relevant subgroup, amplification of *CCND1* had a NPV of 83 %. However, this evidence is based on only one study, and these results will have to be validated in a larger cohort of early OSCC, with subsite analysis. If these results confirm an association between *CCND1* or 11q13 amplification and the presence of occult nodal disease in less than 20 %of the patients, this biomarker is of additional value in deciding as to whether or not treat the neck at early stage OSCC.

## Electronic supplementary material

Below is the link to the electronic supplementary material.

Supplementary Table S1 (DOCX 18 kb)

## Abbreviations

HNSCC: Head and neck squamous cell carcinoma
OSCC: Oral squamous cell carcinoma
LN: Lymph node
LNM: Lymph node metastasis
MRI: Magnetic resonance imaging
OR: Odds ratio
NPV: Negative predictive value
PPV: Positive predictive value
CI: Confidence interval
FISH: Fluorescence in situ hybridization
CGH: Comparative genomic hybridization
IHC: Immunohistochemistry
