# Supplementary material for: The diagnostic value of 11q13 amplification and protein expression in the detection of nodal metastasis from oral squamous cell carcinoma: a systematic review and meta-analysis
Source: Virchows Arch. 2015 Feb 7;466(4):363–73. doi: 10.1007/s00428-015-1719-6 (PMC4392171; doi:10.1007/s00428-015-1719-6)
Supplement: Supplementary file 1 — (DOCX 18 kb) [file 428_2015_1719_MOESM1_ESM.docx]

| **Supplementary Table 1.** Search query for systematic review | |
| --- | --- |
| *Database* | *Search query April 2014* |
| PubMed | (survival[Title/Abstract] OR OS[Title/Abstract] OR DSS[Title/Abstract] OR DFS[Title/Abstract] OR prognosis[Title/Abstract] OR prognostic[Title/Abstract] OR metastasis[Title/Abstract] OR metastases[Title/Abstract] OR nodal[Title/Abstract] OR “lymph node”[Title/Abstract] OR “lymph nodes”[Title/Abstract] OR LN[Title/Abstract] OR LNM[Title/Abstract] OR Neoplasm Metastasis[MeSH Terms] OR Prognosis[MeSH Terms]) AND (((“head neck”[Title/Abstract] OR “head and neck”[Title/Abstract] OR oral[Title/Abstract] OR tongue[Title/Abstract] OR mouth[Title/Abstract] OR buccal[Title/Abstract] OR oropharyngeal[Title/Abstract] OR pharyngeal[Title/Abstract] OR pharynx[Title/Abstract] OR oropharynx[Title/Abstract]) AND (SCC[Title/Abstract] OR SCCs[Title/Abstract] OR oncology[Title/Abstract] OR oncological[Title/Abstract] OR malignant[Title/Abstract] OR malignance[Title/Abstract] OR cancerous[Title/Abstract] OR cancer[Title/Abstract] OR cancers[Title/Abstract] OR carcinoma[Title/Abstract] OR carcinomas[Title/Abstract] OR neoplasm[Title/Abstract] OR neoplasms[Title/Abstract] OR malign[Title/Abstract] OR malignancy[Title/Abstract] OR malignancies[Title/Abstract] OR tumor[Title/Abstract] OR tumors[Title/Abstract] OR tumour[Title/Abstract] OR tumours[Title/Abstract])) OR OSCC[Title/Abstract] OR HNSCC[Title/Abstract] OR OPSCC[Title/Abstract] OR head and neck neoplasms[MeSH Terms]) AND (11q13[Title/Abstract] OR 11q13.3[Title/Abstract] OR CPT1A[Title/Abstract] OR "carnitine palmitoyltransferase 1a"[Title/Abstract] OR CPT1[Title/Abstract] OR CPT1-L[Title/Abstract] OR L-CPT1[Title/Abstract] OR MRPL21[Title/Abstract] OR "mitochondrial ribosomal protein L21"[Title/Abstract] OR L21mt[Title/Abstract] OR MRP-L21[Title/Abstract] OR IGHMBP2[Title/Abstract] OR "immunoglobulin mu binding protein 2"[Title/Abstract] OR HCSA[Title/Abstract] OR HMN6[Title/Abstract] OR CATF1[Title/Abstract] OR SMARD1[Title/Abstract] OR SMUBP2[Title/Abstract] OR ZFAND7[Title/Abstract] OR MRGPRD[Title/Abstract] OR "MAS-related GPR member D"[Title/Abstract] OR MRGD[Title/Abstract] OR TGR7[Title/Abstract] OR MRGPRF[Title/Abstract] OR "MAS-related GPR member F"[Title/Abstract] OR RTA[Title/Abstract] OR MRGF[Title/Abstract] OR GPR140[Title/Abstract] OR GPR168[Title/Abstract] OR TPCN2[Title/Abstract] OR "two pore segment channel 2"[Title/Abstract] OR TPC2[Title/Abstract] OR SHEP10[Title/Abstract] OR MYEOV[Title/Abstract] OR "myeloma overexpressed"[Title/Abstract] OR OCIM[Title/Abstract] OR LOC390218[Title/Abstract] OR IFITM9P[Title/Abstract] OR "interferon induced transmembrane protein 9 pseudogene"[Title/Abstract] OR LOC399919[Title/Abstract] OR CCND1[Title/Abstract] OR cyclin D1[Title/Abstract] OR PRAD1[Title/Abstract] OR "parathyroid adenomatosis 1"[Title/Abstract] OR BCL1[Title/Abstract] OR U21B31[Title/Abstract] OR D11S287E[Title/Abstract] OR FLJ42258[Title/Abstract] OR ORAOV1[Title/Abstract] OR "oral cancer overexpressed 1"[Title/Abstract] OR TAOS1[Title/Abstract] OR FGF19[Title/Abstract] OR "fibroblast growth factor 19"[Title/Abstract] OR FGF4[Title/Abstract] OR "fibroblast growth factor 4"[Title/Abstract] OR HST[Title/Abstract] OR KFGF[Title/Abstract] OR HST-1[Title/Abstract] OR HSTF1[Title/Abstract] OR K-FGF[Title/Abstract] OR HBGF-4[Title/Abstract] OR FGF3[Title/Abstract] OR "fibroblast growth factor 3"[Title/Abstract] OR INT2[Title/Abstract] OR HBGF-3[Title/Abstract] OR LOC399920[Title/Abstract] OR TMEM16A[Title/Abstract] OR "transmembrane protein 16a"[Title/Abstract] OR TAOS2[Title/Abstract] OR ANO1[Title/Abstract] OR "anoctamin 1"[Title/Abstract] OR DOG1[Title/Abstract] OR ORAOV2[Title/Abstract] OR FADD[Title/Abstract] OR "fas associated via death domain"[Title/Abstract] OR "fas tnfrsf6 associated via death domain"[Title/Abstract] OR GIG3[Title/Abstract] OR MORT1[Title/Abstract] OR PPFIA1[Title/Abstract] OR LIP1[Title/Abstract] OR "LIP.1"[Title/Abstract] OR LIPRIN[Title/Abstract] OR CTTN[Title/Abstract] OR cortactin[Title/Abstract] OR EMS1[Title/Abstract] OR SHANK2[Title/Abstract] OR "SH3 and multiple ankyrin repeat domains 2"[Title/Abstract] OR SHANK[Title/Abstract] OR AUTS17[Title/Abstract] OR CORTBP1[Title/Abstract] OR CTTNBP1[Title/Abstract] OR ProSAP1[Title/Abstract] OR SPANK-3[Title/Abstract] OR LOC399921[Title/Abstract]) |
| EMBASE | (survival:ti,ab OR OS:ti,ab OR DSS:ti,ab OR DFS:ti,ab OR prognosis:ti,ab OR prognostic:ti,ab OR metastasis:ti,ab OR metastases:ti,ab OR nodal:ti,ab OR ‘lymph node’:ti,ab OR ‘lymph nodes’:ti,ab OR LN:ti,ab OR LNM:ti,ab OR ‘metastasis’ OR ‘prognosis’) AND (((‘head neck’:ti,ab OR ‘head and neck’:ti,ab OR oral:ti,ab OR tongue:ti,ab OR mouth:ti,ab OR buccal:ti,ab OR oropharyngeal:ti,ab OR pharyngeal:ti,ab OR pharynx:ti,ab OR oropharynx:ti,ab) AND (SCC:ti,ab OR SCCs:ti,ab OR oncology:ti,ab OR oncological:ti,ab OR malignant:ti,ab OR malignance:ti,ab OR cancerous:ti,ab OR cancer:ti,ab OR cancers:ti,ab OR carcinoma:ti,ab OR carcinomas:ti,ab OR neoplasm:ti,ab OR neoplasms:ti,ab OR malign:ti,ab OR malignancy:ti,ab OR malignancies:ti,ab OR tumor:ti,ab OR tumors:ti,ab OR tumour:ti,ab OR tumours:ti,ab)) OR OSCC:ti,ab OR HNSCC:ti,ab OR OPSCC:ti,ab OR ‘head neck tumor’) AND (11q13:ti,ab OR 11q13.3:ti,ab OR CPT1A:ti,ab OR ‘carnitine palmitoyltransferase 1a’:ti,ab OR CPT1:ti,ab OR CPT1-L:ti,ab OR L-CPT1:ti,ab OR MRPL21:ti,ab OR ‘mitochondrial ribosomal protein L21’:ti,ab OR L21mt:ti,ab OR MRP-L21:ti,ab OR IGHMBP2:ti,ab OR ‘immunoglobulin mu binding protein 2’:ti,ab OR HCSA:ti,ab OR HMN6:ti,ab OR CATF1:ti,ab OR SMARD1:ti,ab OR SMUBP2:ti,ab OR ZFAND7:ti,ab OR MRGPRD:ti,ab OR ‘MAS-related GPR member D’:ti,ab OR MRGD:ti,ab OR TGR7:ti,ab OR MRGPRF:ti,ab OR ‘MAS-related GPR member F’:ti,ab OR RTA:ti,ab OR MRGF:ti,ab OR GPR140:ti,ab OR GPR168:ti,ab OR TPCN2:ti,ab OR ‘two pore segment channel 2’:ti,ab OR TPC2:ti,ab OR SHEP10:ti,ab OR MYEOV:ti,ab OR ‘myeloma overexpressed’:ti,ab OR OCIM:ti,ab OR LOC390218:ti,ab OR IFITM9P:ti,ab OR ‘interferon induced transmembrane protein 9 pseudogene’:ti,ab OR LOC399919:ti,ab OR CCND1:ti,ab OR cyclin D1:ti,ab OR PRAD1:ti,ab OR ‘parathyroid adenomatosis 1’:ti,ab OR BCL1:ti,ab OR U21B31:ti,ab OR D11S287E:ti,ab OR FLJ42258:ti,ab OR ORAOV1:ti,ab OR ‘oral cancer overexpressed 1’:ti,ab OR TAOS1:ti,ab OR FGF19:ti,ab OR ‘fibroblast growth factor 19’:ti,ab OR FGF4:ti,ab OR ‘fibroblast growth factor 4’:ti,ab OR HST:ti,ab OR KFGF:ti,ab OR HST-1:ti,ab OR HSTF1:ti,ab OR K-FGF:ti,ab OR HBGF-4:ti,ab OR FGF3:ti,ab OR ‘fibroblast growth factor 3’:ti,ab OR INT2:ti,ab OR HBGF-3:ti,ab OR LOC399920:ti,ab OR TMEM16A:ti,ab OR ‘transmembrane protein 16a’:ti,ab OR TAOS2:ti,ab OR ANO1:ti,ab OR ‘anoctamin 1’:ti,ab OR DOG1:ti,ab OR ORAOV2:ti,ab OR FADD:ti,ab OR ‘fas associated via death domain’:ti,ab OR ‘fas tnfrsf6 associated via death domain’:ti,ab OR GIG3:ti,ab OR MORT1:ti,ab OR PPFIA1:ti,ab OR LIP1:ti,ab OR ‘LIP.1’:ti,ab OR LIPRIN:ti,ab OR CTTN:ti,ab OR cortactin:ti,ab OR EMS1:ti,ab OR SHANK2:ti,ab OR ‘SH3 and multiple ankyrin repeat domains 2’:ti,ab OR SHANK:ti,ab OR AUTS17:ti,ab OR CORTBP1:ti,ab OR CTTNBP1:ti,ab OR ProSAP1:ti,ab OR SPANK-3:ti,ab OR LOC399921:ti,ab) |
| Cochrane Library | (survival OR OS OR DSS OR DFS OR prognosis OR prognostic OR metastasis OR metastases OR nodal OR ‘lymph node’ OR ‘lymph nodes’ OR LN OR LNM) AND (((‘head neck’ OR ‘head and neck’ OR oral OR tongue OR mouth OR buccal OR oropharyngeal OR pharyngeal OR pharynx OR oropharynx) AND (SCC OR SCCs OR oncology OR oncological OR malignant OR malignance OR cancerous OR cancer OR cancers OR carcinoma OR carcinomas OR neoplasm OR neoplasms OR malign OR malignancy OR malignancies OR tumor OR tumors OR tumour OR tumours)) OR OSCC OR HNSCC OR OPSCC) AND (11q13 OR 11q13.3 OR CPT1A OR ‘carnitine palmitoyltransferase 1a’ OR CPT1 OR CPT1-L OR L-CPT1 OR MRPL21 OR ‘mitochondrial ribosomal protein L21’ OR L21mt OR MRP-L21 OR IGHMBP2 OR ‘immunoglobulin mu binding protein 2’ OR HCSA OR HMN6 OR CATF1 OR SMARD1 OR SMUBP2 OR ZFAND7 OR MRGPRD OR ‘MAS-related GPR member D’ OR MRGD OR TGR7 OR MRGPRF OR ‘MAS-related GPR member F’ OR RTA OR MRGF OR GPR140 OR GPR168 OR TPCN2 OR ‘two pore segment channel 2’ OR TPC2 OR SHEP10 OR MYEOV OR ‘myeloma overexpressed’ OR OCIM OR LOC390218 OR IFITM9P OR ‘interferon induced transmembrane protein 9 pseudogene’ OR LOC399919 OR CCND1 OR cyclin D1 OR PRAD1 OR ‘parathyroid adenomatosis 1’ OR BCL1 OR U21B31 OR D11S287E OR FLJ42258 OR ORAOV1 OR ‘oral cancer overexpressed 1’ OR TAOS1 OR FGF19 OR ‘fibroblast growth factor 19’ OR FGF4 OR ‘fibroblast growth factor 4’ OR HST OR KFGF OR HST-1 OR HSTF1 OR K-FGF OR HBGF-4 OR FGF3 OR ‘fibroblast growth factor 3’ OR INT2 OR HBGF-3 OR LOC399920 OR TMEM16A OR ‘transmembrane protein 16a’ OR TAOS2 OR ANO1 OR ‘anoctamin 1’ OR DOG1 OR ORAOV2 OR FADD OR ‘fas associated via death domain’ OR ‘fas tnfrsf6 associated via death domain’ OR GIG3 OR MORT1 OR PPFIA1 OR LIP1 OR ‘LIP.1’ OR LIPRIN OR CTTN OR cortactin OR EMS1 OR SHANK2 OR ‘SH3 and multiple ankyrin repeat domains 2’ OR SHANK OR AUTS17 OR CORTBP1 OR CTTNBP1 OR ProSAP1 OR SPANK-3 OR LOC399921) |
